# Recent advances in the medical treatment of breast cancer

**DOI:** 10.12688/f1000research.9619.1

**Published:** 2016-11-29

**Authors:** Daniel A. Vorobiof

**Affiliations:** 1 Sandton Oncology Centre, Sandton, Johannesburg, South Africa

**Keywords:** breast cancer, chemotherapeutic drugs, breast cancer therapeutics, endocrine-dependent breast cancer, HER2-positive breast cancer, metastatic disease

## Abstract

Over the past few decades, the systemic therapy of breast cancer (early and advanced) has changed considerably. For the past 40–50 years, and since the discovery and further therapeutic use of tamoxifen, a selective estrogen receptor modulator, breast cancer treatment has become the model for the development and success of tailored medical treatment. Much still needs to be done in improving outcomes for all patients with breast cancer, and especially for those who have advanced breast cancer, a challenging area for medical oncologists. Ongoing international clinical trials are currently evaluating new therapeutic approaches and identifying specific biological subsets that could determine a patient’s ability to respond to particular chemotherapeutic drugs.

## Introduction

The worldwide incidence of breast cancer continues to increase, and approximately 1.7 million new cases are diagnosed yearly. It also remains a leading cause of death with approximately 520,000 deaths/year, as reported by the World Health Organization in an updated breast cancer fact sheet of 2015 ^1^. Therapeutic approaches for breast cancer have changed over the past few decades, and the use of systemic therapy for early and advanced disease tailored to the individual patient holds the promise of delivering treatment to those in need and who could benefit the most. In this report, emerging therapeutic options for patients with endocrine-dependent breast cancer, monoclonal antibodies for those with HER2-positive disease, and newer available systemic chemotherapeutic agents will be discussed.

## Endocrine-dependent breast cancer

Hormonal therapy for those patients with breast cancer expressing the endocrine receptors estrogen and progesterone has been utilized for longer than 30 years with the administration of tamoxifen, a selective estrogen receptor modulator (SERM) and probably the first targeted therapy widely used in cancer treatment ^2^. The options for the treatment of hormone-sensitive breast cancer have expanded in recent years with a number of other effective endocrine agents that reduce estrogen biosynthesis: the aromatase inhibitors (AIs) such as letrozole, exemestane, and anastrozole, gonadotropin-releasing hormone agonists (GnRH) such as goserelin and leuprolide, and selective estrogen receptor downregulators (SERDs) such as fulvestrant. Since the use of these compounds, alone or in combination, a substantial improvement in prolongation of disease-free intervals and survival outcomes has been reported.

Recently, two international multicentric clinical trials reported the continuous use of tamoxifen or an AI for a prolonged period of time (10 years) for early breast cancer endocrine-positive patients ^3, 4^. In the ATLAS (adjuvant tamoxifen: longer against shorter) randomized trial ^3^, nearly 13,000 women with early breast cancer who completed 5 years of treatment with tamoxifen were randomized to continue tamoxifen for a total of 10 years or to stop at 5 years. The results obtained showed that for those patients continuing tamoxifen to 10 years, a further reduction in recurrence and mortality happened, in particular after year 10. These results, according to the ATLAS Collaborative Group investigators, suggest that 10 years of tamoxifen treatment can approximately halve breast cancer mortality during the second decade after diagnosis.

Another recently published study (MA17R) ^4^ extended treatment with a non-steroidal AI (letrozole) to 10 years for postmenopausal endocrine-positive early breast cancer patients. Patients who received 5 years with an AI monotherapy upfront or after tamoxifen therapy were randomized to an extra 5 years. More than 1,900 patients were enrolled, and the extension of the AI to 10 years resulted in significantly higher rates of disease-free survival and lower incidence of contralateral breast cancer. As expected, bone-related side effects occurred more frequently among patients receiving letrozole than placebo.

These longer tamoxifen and AI prospective clinical studies, together with earlier published clinical trials, confirmed that women at a higher risk of developing recurrence or metastatic disease should strongly consider continuation of their adjuvant therapy to 10 years without any long-term worsening of their quality of life.

Despite hormonal standard therapy, approximately 20–30% of patients with endocrine-responsive breast cancer will suffer recurrences and the development of metastatic disease as they experience a biochemical mechanism of endocrine resistance. Recent ASCO guidelines on endocrine therapy for advanced breast cancer reaffirm many of the principles that should be considered when making treatment decisions in this patient population ^5^.

Significant progress has been made recently in this area, and newer therapeutic targets have been developed against a number of hormonal resistance mechanisms. Inhibitors of these pathways are under development to improve the efficacy of hormonal therapy, and some have already become FDA approved and commercially available.

It is known that resistance to endocrine therapy, and to some chemotherapy drugs, is usually associated with a number of different molecular mechanisms. PI3K/AKT/mTOR is the most commonly altered pathway in hormonal-positive breast cancer, and there are a number of agents targeting this specific pathway ^5– 9^. Everolimus, together with a steroidal AI (exemestane), has shown promising benefits in international clinical trials. It is FDA approved and available around the world. The regulatory approval was based on data from the Bolero trials, of which Bolero 2 has been completed. Based on doubling of the progression-free survival and prolongation of survival (albeit not statistically significant), the FDA approved it 6– 9.

Pan class PI3K inhibitors such as buparlisib, pictilisib, alpelisib, and taselisib continue to be investigated, as they hold the potential to overcome hormone resistance and improve responses. Promising activity has already been shown in the clinic, but there are some limitations, mainly due to dose-limiting toxicities (including skin rash, diarrhea, and others) ^10^.

*De novo* or acquired resistance to adjuvant therapy and metastatic breast cancer remains an important therapeutic challenge. Cyclin D1 interacts with cyclin-dependent kinases 4 and 6 (CDK 4 and 6) in an active protein complex that promotes cell proliferation. Cyclin D1 is probably the most frequently overexpressed gene in primary breast cancer (approximately 15% of breast cancer patients). Therefore, CDK 4 and 6 represent potential therapeutic targets for endocrine-responsive breast cancer. Inhibition of the CDK 4/6 pathways is possible by small molecule inhibitor drugs such as palbociclib, ribociclib, and abemaciclib ^10– 14^.

Palbociclib was the first anti-CDK 4/6 drug approved by the FDA based on the Paloma clinical trials. Ribociclib has recently been approved (following the results of the MONALEESA trials). Abemaciclib has recently been granted breakthrough therapy designation by the FDA. This was based on data of an early clinical trial (MONARCH 1) that confirmed the efficacy and safety of abemaciclib in women with advanced or metastatic, refractory hormonal-positive breast cancer. Although the most common grade 4 toxicity is neutropenia (in 5% of patients), it is usually manageable and no febrile neutropenia was reported ^12– 14^. The hope remains that, in the future, reliable biomarkers providing oncologists with objective tools to make the best therapeutic decisions will become commonly available.

## HER2-positive breast cancer

Since the introduction of the first anti-HER2 targeted therapy (trastuzumab) approximately 16 years ago, many other emerging monoclonal antibodies have been investigated in prospective randomized trials. Trastuzumab has significantly increased the survival of breast cancer patients with HER2-positive disease in the neoadjuvant, adjuvant, and metastatic groups of patients ^15, 16^.

Newer drugs such as pertuzumab in combination with trastuzumab and chemotherapy, and TDM-1, an antibody drug conjugate, have shown a proven, consistent benefit with prolongation of disease-free intervals but not always an overall survival benefit. Final results from the CLEOPATRA study showed that the combination of two targeted agents, trastuzumab and pertuzumab, significantly prolonged survival in patients with HER2-positive advanced breast cancer when compared to trastuzumab alone. All patients received chemotherapy with docetaxel together with the targeted drugs ^17^. Different clinical studies in phase II and III have confirmed the value of TDM-1 administration in second- or third-line therapy for advanced breast cancer ^17, 18^.

Neratinib is an oral small tyrosine kinase inhibitor molecule initially tested in patients with HER2-positive metastatic breast cancer that showed antitumor activity in previously treated patients. The ExteNET phase III trial of neratinib versus placebo after adjuvant treatment with trastuzumab accrued more than 2,400 patients. The results demonstrated that neratinib after trastuzumab resulted in a 49% reduction of risk (recurrence or death) with a two-year disease-free interval advantage when compared to placebo. The downside of this drug is the relatively high incidence of side effects (diarrhea, fatigue, nausea, and vomiting). It remains one of the most promising new drugs for HER2-positive breast cancer, and efforts to reduce and control its known side effects will ultimately allow for a safer use of this novel drug ^19^.

Unfortunately, most of these drugs are very costly and because of this they are not readily available worldwide. Efforts to ensure that treatments remain cost effective in environments of limited resources should be implemented. An alternative solution is to develop a cheaper, equally effective alternative, a biosimilar agent. Biosimilars are not biochemically identical to the original and must be tested in clinical trials to ensure that they are not inferior (in biologic activity, safety, and efficacy) ^20– 23^.

A recent study performed with MYL-14010, a trastuzumab biosimilar, was presented at ASCO 2016. The clinical trial (HERITAGE) was a double blind, prospective randomized trial that enrolled over 450 patients with HER2-positive advanced breast cancer and who never received prior chemotherapy or an anti-HER2 agent for their metastatic disease. The conclusion by the investigators was that an equivalent efficacy was demonstrated between the biosimilar MYL-14010 and trastuzumab, with similar safety, immunogenicity, and pharmacokinetics ^23^.

## Chemotherapy and combinations for metastatic disease

For those patients with advanced HER2-positive breast cancer, the treatment scenario is evolving rapidly as many effective targeted therapies are being developed and combinations of drugs are shown to induce a higher benefit.

For patients with HER2-negative metastatic disease, the treatment approach should be tailored according to a number of prognostic factors, which include endocrine tumor status, other possible actionable targets (androgen receptor, BRCA mutation), hormonal status, prior therapies (adjuvant and metastatic), disease-free interval, comorbidities, performance status, burden of metastatic disease, patient preferences, prior toxicities, geographic accessibility to the closest cancer treatment center, and available as well as affordable cancer therapies.

Over the past few years, the International Consensus Conference for Advanced Breast Cancer (ABC) ^24^ has established itself as a major international developer of guidelines in the treatment of advanced disease based on the most up-to-date evidence that can be used worldwide and in different healthcare settings. The new consensus guidelines will be published during the last quarter of this year.

## Immunotherapy

During the last few years, immunotherapy has been shown to induce remarkable responses in the treatment of advanced malignant melanoma, non-small-cell lung cancer, and bladder cancer. Several new drugs (anti-CTLA4 and anti-PD-1 and PDL-1) have been approved by the FDA. Clinical trials with this new group of drugs for patients with advanced breast cancer have shown induction of moderate overall response rates. In some subsets of patients (those with triple-negative disease and those expressing PDL-1), an improved clinical activity was seen ^25, 26^. Further investigation of these novel immunotherapy drugs is warranted, and new clinical trials are currently ongoing.

## New genomic testing

Are all patients with early breast cancer benefiting from adjuvant chemotherapy? With this question in mind, global investigators, motivated by the development of new genomic profiling tests (Oncotype Dx and Mammaprint), have been focusing on how to personalize therapies so that only the necessary treatment is delivered. Recent publications have demonstrated how genomic information provided by the use of a 21-gene assay (Oncotype Dx) or a 70-gene assay (Mammaprint), combined with the patient’s clinicopathologic status, can come up with a risk score predicting the need for adjuvant chemotherapy ^27– 29^. The results confirmed that genomic risk assessment can reduce the need for chemotherapy in up to 46% of patients who might otherwise receive it on the basis of their clinical and pathologic assessment alone.

Oncotype Dx and Mammaprint are first-generation genomic signature tests able to better predict early relapses. PAM50 and Endopredict are second-generation genomic signatures helpful in predicting late relapses, guiding clinicians during the 5–10-year follow-up of those patients.

## Conclusion

The advent of new drugs targeting specific actionable targets has led to considerable progress in the treatment of breast cancer over the past few years. Challenges remain, such as resistance to systemic therapy (endocrine and others), high cost of treatments, and limited availability in many countries of appropriate cancer services.

We must continue to find ways to improve our available technology to provide proper guidance for those living with the disease, and for those at high risk of developing it, and to develop new, more effective therapies to substantially improve breast cancer patients’ outcomes worldwide. Tailoring treatments to the individual patient holds the promise of guiding them as they face difficult treatment decisions in an effort to improve their long-term outcomes.
